# A Nationwide Retrospective Analysis of Socioeconomic Factors Associated With Eosinophilic Colitis: A Population-Based Study

**DOI:** 10.1016/j.gastha.2025.100663

**Published:** 2025-03-22

**Authors:** Bibek Karki, Samjhana Belbase, Michelle Bernshteyn, Ajit Brar, Hussain Murtaza, Nikky Maharjan, Navindra Dhakal, Subash Ghimire, Pujan Kandel, Calvin Ghimire, Philip McDonald, Matthew Lincoln, Michael Georgetson

**Affiliations:** 1 Department of Internal Medicine, Hurley Medical Center/MSU, Flint, Michigan; 2 Division of Gastroenterology, Robert Packer Hospital, Sayre, Pennsylvania; 3 Division of Gastroenterology, Sparrow Hospital, Lansing, Michigan; 4 Department of Pediatrics, Burtibang PHC, Baglung, Nepal; 5 Division of Gastroenterology, Citrus Memorial Hospital, Inverness, Florida; 6 Department of Internal Medicine, Princeton Community Hospital, Princeton, West Virginia

**Keywords:** Eosinophilic Gastrointestinal Disorders, Colitis, Prognosis, Disparities

## Abstract

**Background and Aims:**

Eosinophilic colitis (EC) is a rare gastrointestinal inflammation of the colon characterized by eosinophilic infiltration into the colonic wall. The study aimed to assess the socioeconomic factors associated with EC.

**Methods:**

A retrospective cohort study was conducted using the 2016–2020 National Inpatient Sample, including adult patients (≥18 years) admitted with EC.

**Results:**

Among 4353 EC cases identified, males are more likely to be hospitalized than females (51.8% vs 48.2%, *P* < .001). Caucasians consisted of 81.7% of total cases, followed by African Americans (8.1%), Hispanics (6.3%), and Asians (1.3%) (*P* < .001). 50.6% of EC cases had Private/Health Maintenance Organization insurance followed by Medicare (26.5%), Medicaid (15.7%), and 7.2% were uninsured (*P* < .001). Only 16.5% of patients had a Charlson Comorbidity Index ≥3.

**Conclusion:**

Caucasians, males, and those with private insurance were more likely to be diagnosed with EC. The correlation between insurance coverage and hospitalizations shows health-care disparities and implies the need for equitable health-care delivery.

## Introduction

Eosinophilic colitis (EC) is a rare gastrointestinal (GI) condition characterized by eosinophilic infiltration in the colonic tissue.^1^ As a subset of eosinophilic GI disorders, it can affect any part of the GI tract, particularly the esophagus, stomach, and colon. It is further classified by the layer of the tissue involved (mucosal, muscular, or subserosal).^1^, ^2^, ^3^, ^4^ EC exhibits a bimodal distribution, with neonates and young adults more often affected.^3^

While the exact cause of EC is unclear, it may involve various causes, including food allergens, infections (often parasitic), hypereosinophilic syndrome, inflammatory bowel disease, celiac disease, connective tissue disorders, vascular disease (scleroderma, dermatomyositis, and polymyositis), and medications (clozapine, and carbamazepine).^5^^,^^6^ Primary EC is a diagnosis of exclusion, requiring the ruling out of secondary causes.^2^

The disease process is thought to arise from a complex interaction of genetic predisposition, immune dysregulation, and environmental factors (such as pollen), with gut microbiota alterations and compromised intestinal epithelial barrier contributing to the disease development.^7^, ^8^, ^9^ Immune-mediated food antigens (eg cow's milk, soy, wheat) are thought to trigger a TH-2 immune response.^9^^,^^10^ An imbalance in proinflammatory and anti-inflammatory signals leads to eosinophilic activation and recruitment in the colonic tissue via inflammatory mediators such as IL-1, IL-4, IL-5, and tumor necrosis factor-α, where IL-4/IL-5 acts as a driver of eosinophilic recruitment and IL-1/tumor necrosis factor-α-acts as enhancers of endothelial adhesion and transmigration.^10^^,^^11^

EC presents clinically with abdominal pain, diarrhea (bloody or non-bloody), and weight loss, sometimes associated with allergic conditions (asthma, hay fever, and eczema). The presentation is more common in neonates and young adults.^1^^,^^12^ Complications may include cecal volvulus causing intestinal obstruction, intussusception, and perforation.

Treatment includes dietary management, which reduces mucosal eosinophils and improves symptoms, though efficacy depends on patient compliance.^13^ Pharmacologic treatment consists of corticosteroid therapy (eg, prednisolone, budesonide) with subsequent rapid tapering.^14^, ^15^, ^16^ For severe, refractory, or steroid-dependent EC, immunomodulatory drugs (eg, azathioprine or 6-mercaptopurine) might be used.^2^^,^^14^ Other drugs like ketotifen, sodium cromoglycate, and biological agents such as omalizumab and mepolizumab can also be used with varying efficacy.^2^^,^^14^

EC diagnosis is likely under-reported due to its diagnosis of exclusion.^16^ Very few studies have described the demographics of EC. This study aims to investigate the relationship between demographic factors (gender, race, ethnicity, and income) and EC hospitalizations using data from the National Inpatient Sample (NIS) database.

## Methods

### Study Design and Data Source

This retrospective nationwide study utilized data from the NIS database (2016–2020) the largest publicly available database in the United States, containing over 7 million hospital visits, and maintained by the Agency for Healthcare Research and Quality.^17^ This large-scale database is ideal for analyzing rare diseases. The authors were involved in study design, data collection, analysis, and interpretation.

### Settings

The study analyzed data from hospitalized patients across diverse United States settings, including rural, urban, teaching, and nonteaching hospitals, enabling a comprehensive evaluation of health-care disparities.

### Participants

The study included adult patients (≥18 years), admitted with a diagnosis of EC between January 1, 2016, and December 31, 2020. The pediatric population (<18 years), and those with other eosinophilic GI disorders (except EC), were excluded. Cases were identified using the validated International Classification of Diseases, 10th Revision (ICD-10) diagnosis codes specific to EC.

### Study Variables

The study included following variables of interest: (1) gender (male and female), (2) race/ethnicity (Caucasian, African American, Hispanic, and Asian), (3) insurance type (Medicare, Medicaid, private/ Health Maintenance Organization (HMO)), (4) median household income (<$45,999, $46,000–$58,999, and >$59,000), (5) hospital location (south, mid-west, west, northeast), (6) hospital teaching status (urban teaching, urban nonteaching, rural), and (7) Charlson Comorbidity Index (CCI).

### Bias

The study used a large database like NIS to help reduce selection bias and other potential biases related to health-care access disparities. The pediatric population was excluded to avoid any age-related confounding factors. The study used validated ICD-10 diagnosis codes to reduce misclassification bias.

### Study Size

The study size was based on the number of EC cases identified in the NIS database over the study period of 5 years. A large population database was included to ensure the robustness of statistical analysis and sufficient power to determine the significant association with EC.

### Statistical Methods

Descriptive analysis was used to summarize the characteristics of the study population. Categorical variables such as gender, race/ethnicity, insurance type, hospital location, and hospital teaching status were initially presented as percentages and compared using chi-square tests. The CCI was also presented as percentages and analyzed for its distribution. The association between socioeconomic factors and EC was analyzed using chi-square tests, and a *P* value < .05 was considered statistically significant.

### Ethical Considerations

This study used the deidentified data from the NIS database, ensuring patient confidentiality and compliance with privacy regulations. As the data were publicly available and contained no identifiable information, institutional review board approval was not required for our study.

## Results

### Study Population and Sample Characteristics

**Figure 1:**
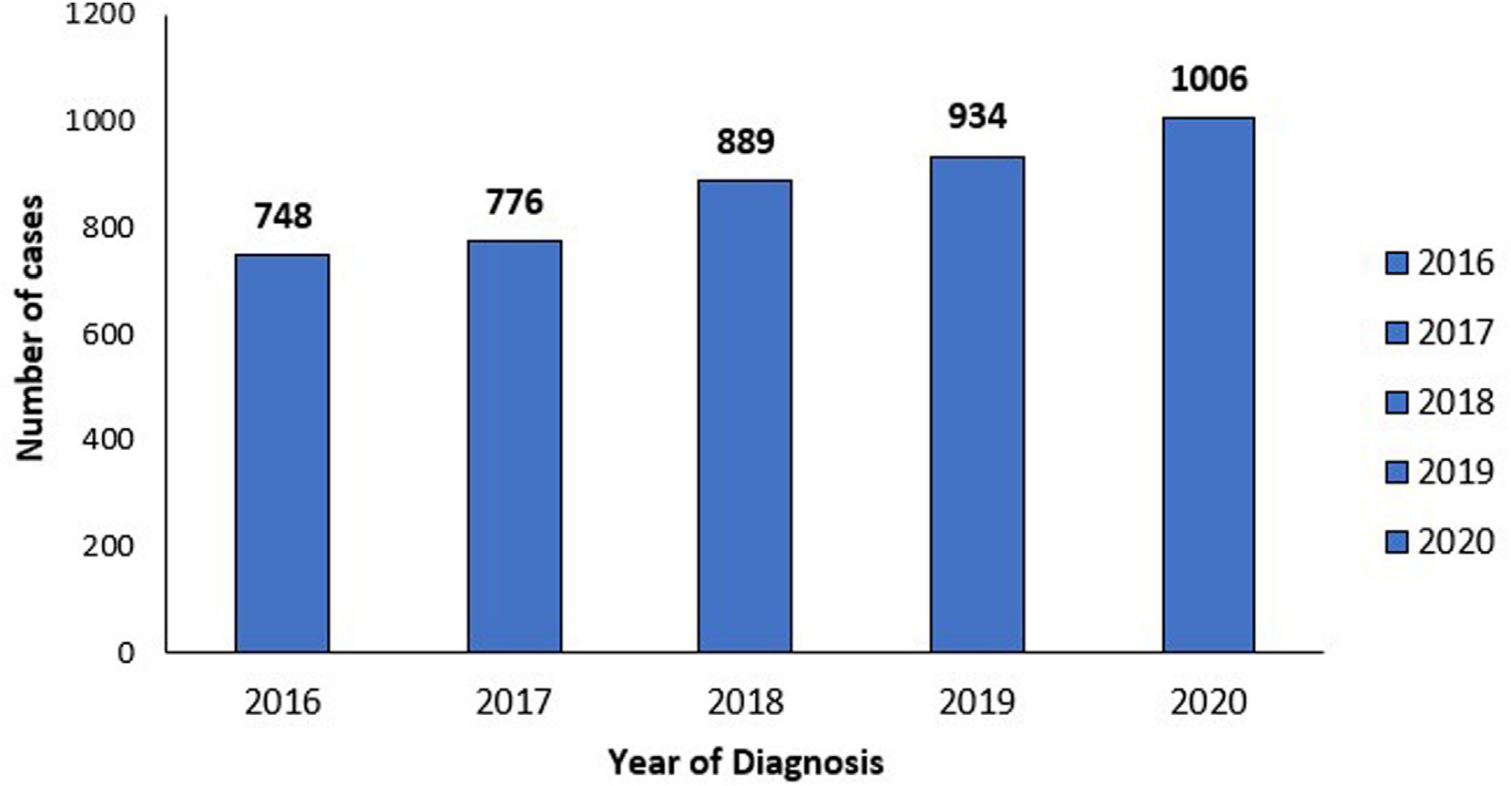
Incidence of EC by year of diagnosis.

Our retrospective study included a total of 4363 cases of EC, using the NIS database during the study period. The number of cases diagnosed annually showed an incremental trend over the 5 years, with 748, 776, 889, 934, and 1006 cases in 2016, 2017, 2018, 2019, and 2020 respectively (Table and Figure 1). There are 10 missing cases of diagnosed EC in terms of year of diagnosis, which could be due to data entry errors, or missing data within the database.

**Table:** Demographic Table of Incidence of EC

Demographic variables	Number of patients (%)
Overall	4363
Gender	
Male	2252 (51.8%)
Female	2099 (48.2%)
Ethnicity	
White	3427 (81.7%)
Black	339 (8.1%)
Hispanic	264 (6.3%)
Asian	55 (1.3%)
Other	110 (2.6%)
Year of diagnosis	
2016	748
2017	776
2018	889
2019	934
2020	1006
Insurance types	
Medicare	1156 (26.5%)
Medicaid	685 (15.7%)
Private/HMO	2207 (50.6%)
Median household income	
<45,999	889 (20.7%)
$46,000–58,999	1011 (23.6%)
>$59,000	2386 (55.6%)
Hospital location	
South	1317 (30.3%)
Midwest	1199 (27.5)
West	1019 (23.4%)
Northeast	818 (18.8%)
Hospital type	
Urban teaching hospitals	3388 (77.8%)
Urban nonteaching	764 (17.6%)
Rural hospitals	201 (4.6%)

### Gender Distribution

**Figure 2:**
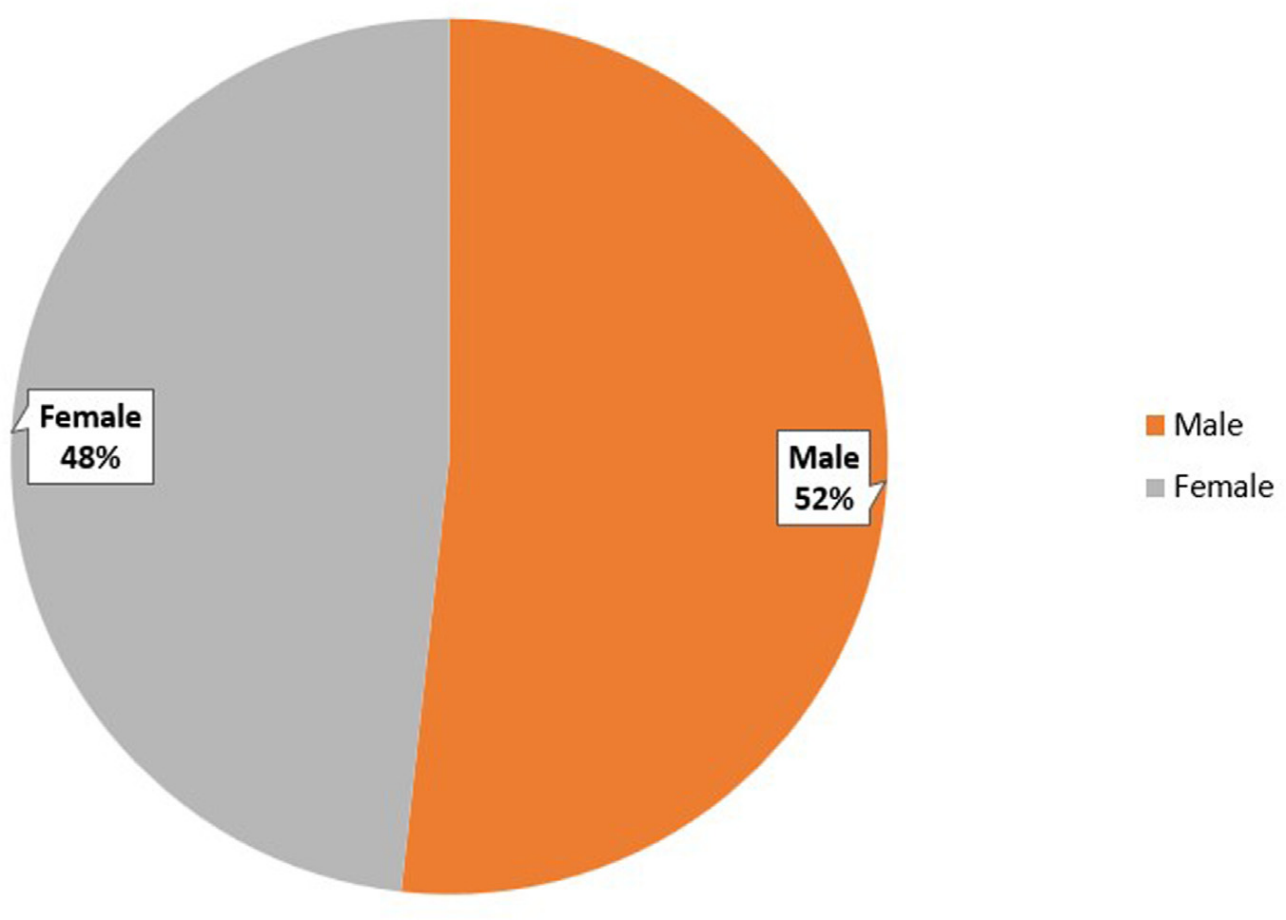
Prevalence of EC based on gender.

Male patients (2,252) accounted for a higher proportion of hospitalizations due to EC compared to females (2,099), (51.8% vs 48.2%, *P* < .001) as shown in Figure 2. There are 12 missing cases in terms of gender distribution, which could be due to incomplete patient records, or anonymization of the identifying gender details due to privacy reasons.

### Race/Ethnicity Distribution

**Figure 3:**
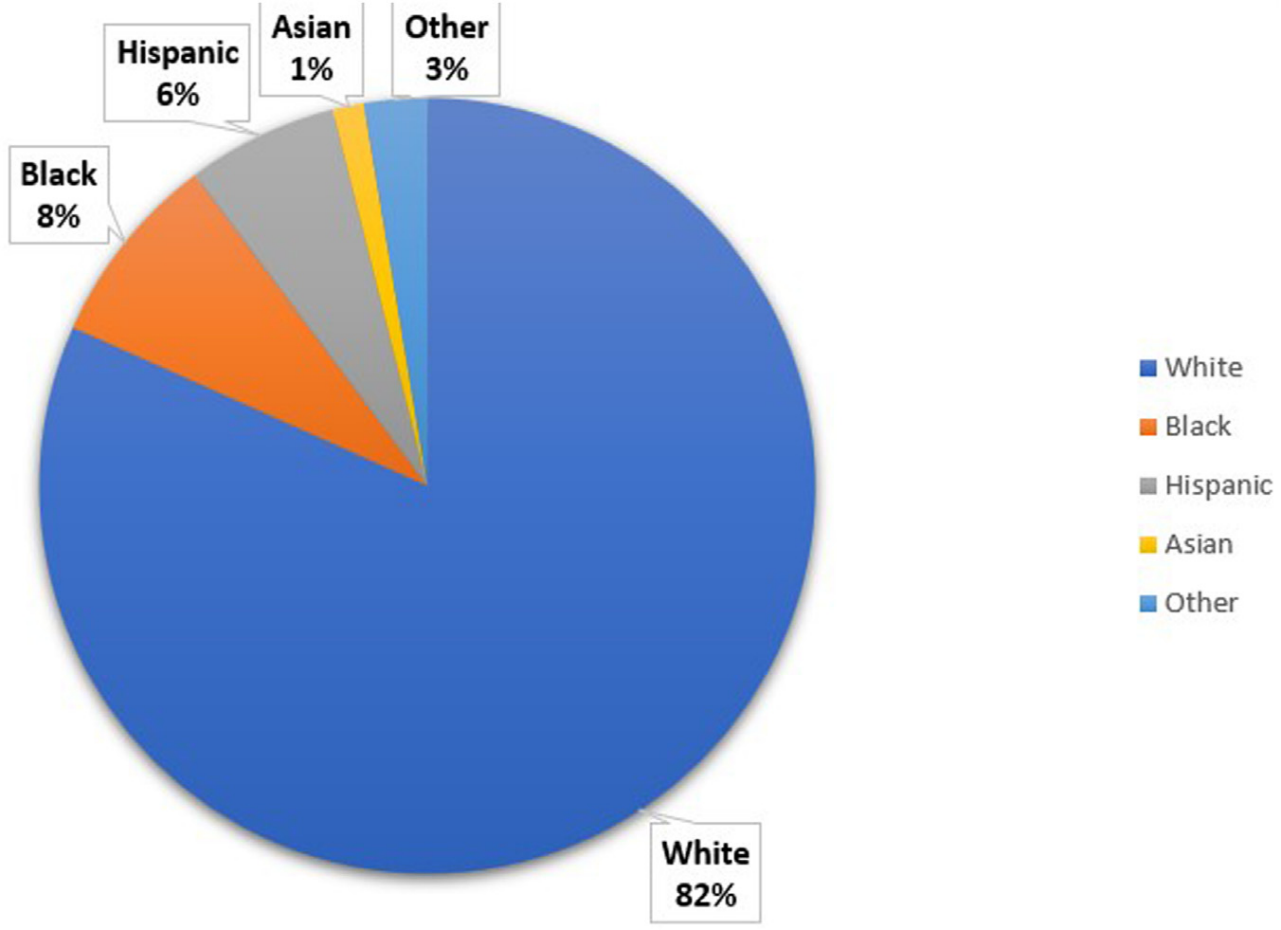
Prevalence of EC based on ethnicity.

Most diagnosed cases of EC were among the Caucasian population, constituting 81.7% (3427) of the cases. In contrast, African American individuals accounted for only 8.1% (339), Hispanics 6.3% (264), Asians 1.3% (55), and other ethnicities 2.6% (110) of the cases (*P* < .001) as shown in Figure 3. There are 168 cases with missing race/ethnic descriptions due to incomplete documentation, data anonymization, patient nondisclosure, and technical issues with standardization of racial/ethnic data collection.

### Median Household Income

**Figure 4:**
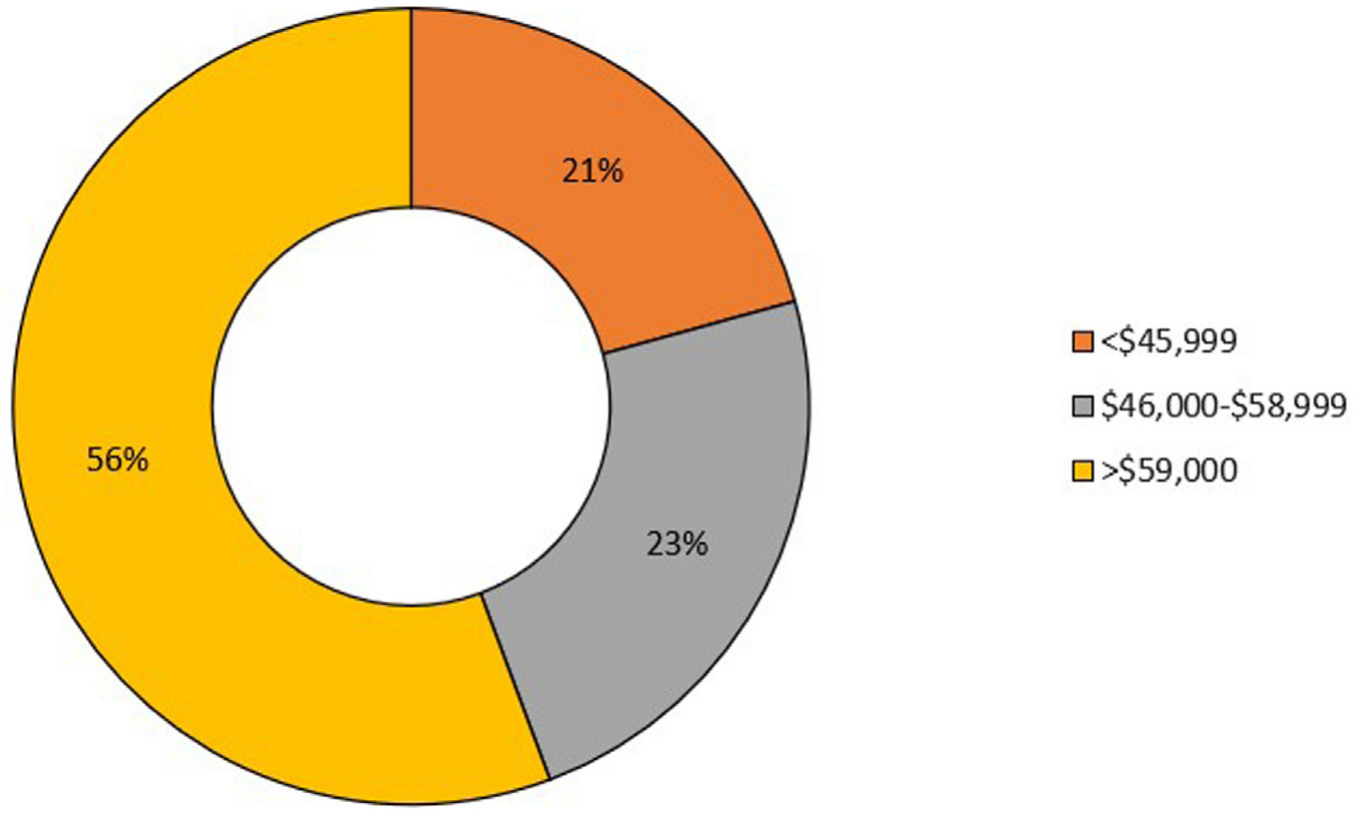
Prevalence of EC based on median household income.

Most of the hospitalized patients with EC had a median household income of $59,000 or more (55.6% of cases), followed by those with a median household income between $46,000 and $58,999 (23.6% of cases), and then those with income of $45,999 or less (20.7% of cases) with *P* value <.001 as shown in Figure 4. There are 77 patients without median household income due to incomplete patient records, and nondisclosure from the patient.

### Hospital Location

**Figure 5:**
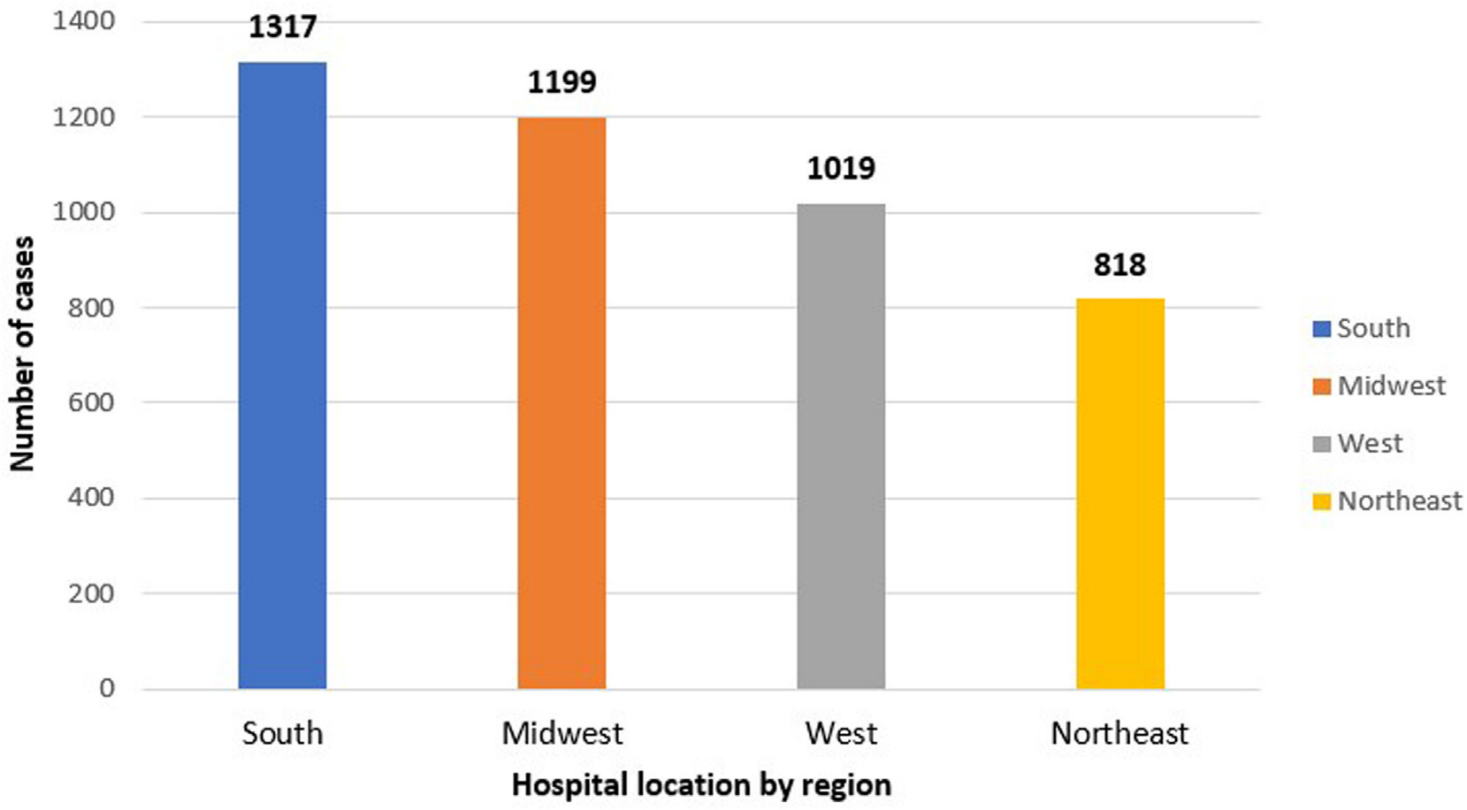
Prevalence of EC based on hospital location.

The highest proportion of reported cases was found in the South (30% of cases), followed by the Midwest (27.5% of cases), the West (23.4% of cases), and the Northeast (18.8% of cases) (*P* < .001) as shown in Figure 5. There are 10 patients with missing hospital locations due to technical errors with data collection.

### Hospital Teaching Status

**Figure 6:**
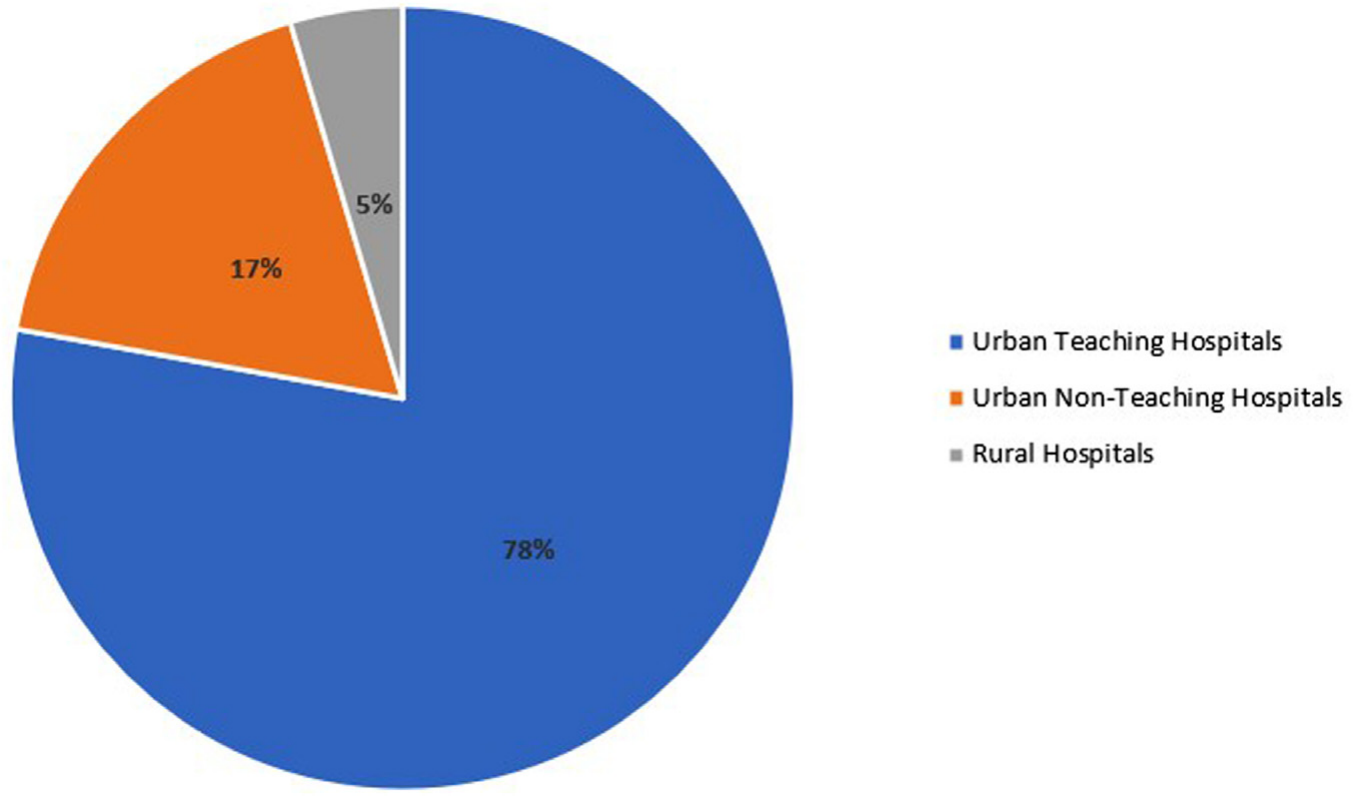
Prevalence of EC based on hospital types.

Hospital type also showed significant variation, with most patients admitted for EC recorded in urban teaching hospitals (77.8%). In comparison, 17.6% of cases were in urban nonteaching hospitals, and 4.6% were in rural hospitals (*P* < .001) as shown in Figure 6. There are 10 missing cases with hospital teaching status likely due to incomplete data reporting, data entry errors, and possibly missing administrative details during data collection.

### Insurance Type

Insurance type showed considerable variability among EC patients. 26.5% had Medicare, 15.7% had Medicaid, and 50.6% had private/HMO insurance coverage (*P* < .001). There are 315 cases with missing insurance information likely due to no insurance for some individuals, and data collection errors.

### Comorbidity Status

Finally, the incidence of EC was based on the CCI to assess comorbidities. Only 16.5% of the study population with EC patients had a CCI of 3 or higher.

## Discussion

Our study provides an overview of the epidemiology of EC, identifying the trends in gender, race, and socioeconomic factors that may influence disease prevalence, as shown in the Supplemental Appendix. The increasing trajectory in diagnosed cases through the 5-year study indicates improved recognition and diagnosis of the condition. However, the COVID-19 pandemic in 2019–2020 may have led to underdiagnosis in 2020, as health-care resources were diverted to pandemic-related care.

Moreover, the gender imbalance observed in hospitalizations attributable to EC, with a greater representation of cases among males, prompts inquiry into potential gender-specific risk factors or genetic predispositions which would necessitate further research. Similarly, the significant ethnic variation in EC cases, with the highest prevalence among Caucasian individuals, suggests the potential racial or genetic factors contributing to disease manifestation.

The correlation between insurance coverage and hospitalizations with EC underscores the pertinent health-care access disparities among diverse patient cohorts, and the importance of targeted interventions to ensure equitable health-care delivery. The use of insurance types also varies with the patient's demographics, such as age, socioeconomic status, and employment. For example, older adults often use Medicare, low-income individuals use Medicaid, and those with higher income or employment use private or HMO insurance. Individuals with private or HMO insurance tend to have more comprehensive coverage to their specialized needs, compared to other insurance types. Hence, EC is more commonly diagnosed among patients with private or HMO insurance.

The correlation between EC cases and median household income is also noteworthy, possibly indicating varied factors such as differential health-care access, dietary habits, and lifestyle choices among individuals with different socioeconomic statuses. Further research is warranted to understand the specific underlying reasons behind this relationship.

Our study indicates the geographical variations in EC prevalence. Identifying such regional patterns can assist health-care providers in tailoring disease management strategies to the specific needs of different regions. The higher incidence of EC cases in urban teaching hospitals may reflect the centers' referral patterns and specialized expertise in managing complex cases. However, this finding also raises questions about potential disparities in access to specialized care, particularly for patients residing in rural areas. Telemedicine and outreach initiatives could be considered to improve access to quality care for patients in underserved regions.^18^

Our study reveals a relatively low CCI among EC patients. This suggests that EC may often be an isolated condition rather than being significantly associated with other comorbidities. Traditionally, EC has been considered a diagnosis of exclusion, necessitating ruling out other potential causes of colitis.^1^^,^^2^^,^^16^ However, our findings challenge the notion that the occurrence of EC is more frequent without the comorbid status than what is mentioned in the literature. However, further research is required to explore the potential associations between EC and other medical conditions that may not be fully captured by the CCI. Understanding these associations could potentially improve the diagnostic approach and improve patient outcomes.

Interesting parallels can be drawn from developing prevalence trends in autism spectrum disorder (ASD) over the years.^19^ Many early studies demonstrated an increased prevalence of ASD in richer communities and caucasian populations.^19^^,^^20^ This relationship is now attributed to higher detection rates and access to more resources in those subsets of the population. Current data reflect that ASD prevalence is higher in people with lower socioeconomic status and lesser education (20). Recent data showcase higher rates of ASD in Hispanic, African American, and Asian Americans highlighting the impact structural inequity places on incidence and prevalence rates.^21^

Current literature lacks comprehensive data elucidating the impact that access to health care, social needs, income, location, and race has had on people suffering from EC. Current data on the disease is mostly sourced from case series and literature reviews. Even fewer studies have provided standardized estimated prevalence data and nationally collected data is lacking.^1^

The study uses a longitudinal approach to provide a temporal trend in the incidence of EC with 5-year study results.

Our study on EC is a retrospective study with the largest patient population providing a wide range of socioeconomic factors that could contribute to the risk factors and diagnosis of EC. Our large study included a patient population of different races/ethnicities, regions, or with various socioeconomic status, which enhances the reliability of the findings, and applicability to a larger population and various regions improving the generalizability of the study results.

Despite the valuable insights gained from this study, some limitations should be acknowledged. Firstly, this study relies on data from the NIS database, and the accuracy of diagnoses and coding may vary across different institutions.^22^ Interestingly, there could be the ambiguity of the ICD coding as the diagnosis of EC with other allergic GI disorders of infancy, but we excluded the pediatric population in our study.^22^ There is also a need for an improvement in coding practices and data collection methods to minimize the missing data in databases and strategies.

Secondly, the database may not capture certain patient-specific variables (diet, alcohol consumption, physical activity, or exposure to allergens) and lifestyle factors (like stress, occupational hazards, and duration of symptom onset to the diagnosis) that could be relevant to EC risk. The preponderance of diagnosis in certain subgroups in contrast to underdiagnosis in other groups may be due to unmet needs in those communities. These factors could account for confounding effects on the onset of EC, which might skew the results. Our study also does not provide data on the treatment modalities or adherence to the treatment and health-seeking behaviors. Lastly, the study's observational nature prevents the establishment of causal relationships between the identified variables and EC incidence.^23^

There could be other potential biases in our study project. The NIS database does not distinguish the readmission of the same disease as a single patient. Hence, the reported number of cases could be an overestimation of EC cases. The research focused only on EC admitted to the hospitals relying only on the NIS database, which might not capture all cases of EC and could lack generalizability. Our observational study doesn't show causality and cannot represent the causal relationships within the defined variables. EC is a diagnosis of exclusion, hence providing variability in the diagnosis.^1^^,^^2^^,^^16^ The higher prevalence in urban teaching hospitals could reflect the referral cases rather than a true correlation with geographical prevalence, skewing the regional disparities. Also, the association between the type of insurance and socioeconomic status may show the disparities in access to health care rather than the correlation to disease prevalence.

The observed gender and racial/ethnic disparities call for a need for further research to investigate underlying genetic or environmental factors leading to the disease prevalence. The association with the prevalence of EC with median household income may reflect differences in health-care access, and environmental exposures. Individuals with private/HMO insurance have comprehensive coverage to have better access to diagnosis and treatment. The predominance of cases in urban teaching hospitals highlights the potential disparities in health-care access compared to those in rural areas. Moreover, the observed disparities in hospitalizations based on gender, ethnicity, insurance type, household income, hospital location, and types underscore the importance of addressing health-care inequities to ensure optimal patient outcomes. Further investigations are warranted to investigate the underlying factors contributing to these disparities and to identify potential interventions for improving patient care and outcomes.

Our study highlights the need for prospective studies to investigate the incidence trend and explore the association with demographic factors over the years. There is an opportunity to investigate the underlying biological, genetic, and environmental mechanisms related to EC. The health-care policy should ensure equitable care by reducing disparities due to health-care access and insurance coverage for all patients with EC. By addressing these implications and conducting research in future directions, health-care professionals can improve their understanding of the disease correlation, diagnosis, and management of EC, thus improving patient outcomes.

## Conclusion

This study provides significant insight into the demographics and epidemiology of EC cases using a large-scale database. The findings may guide further research and help health-care professionals develop targeted strategies for the prevention, diagnosis, and management of EC. Lack of clinical guidelines, clear diagnostic criteria, and a poor understanding of the pathophysiology of EC complicate its initial diagnosis.^1^^,^^16^ Due to the nature of the disease, cases may go undiagnosed for an extended period and there is a hidden burden of disease that may surface in the form of avoidable complications like stricture.

## Figures and Tables

**Supplementary Material:**
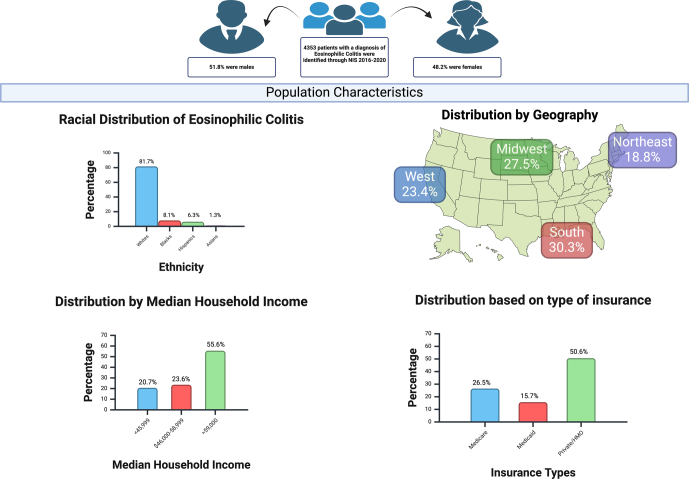
Overview of population characteristics of eosinophilic colitis.
